# How music may support perinatal mental health: an overview

**DOI:** 10.1007/s00737-021-01178-5

**Published:** 2021-08-28

**Authors:** Katie Rose M. Sanfilippo, Lauren Stewart, Vivette Glover

**Affiliations:** 1 Department of Psychology, Goldsmiths, University of London, London, UK; 2 Institute of Reproductive and Developmental Biology, Imperial College London, London, UK

**Keywords:** Music, Singing, Prenatal, Postnatal, Mental health

## Abstract

There is strong evidence that engaging with music can improve our health and well-being. Music-based interventions, approaches and practices, such as group music-making (singing or playing musical instruments), listening to music and music therapy, have all been shown to reduce symptoms of depression and anxiety. Although the existing literature needs expanding, mounting evidence suggests that music-based interventions, approaches and practices may help support maternal mental health prenatally and postnatally. The purpose of this descriptive overview is to provide a broad view of this area by bringing together examples of research across different practices and research disciplines. Selected evidence is examined, showing how music-based interventions, approaches and practices can reduce labour anxiety and pain, anxiety symptoms in pregnancy, postnatal depression symptoms and support maternal-infant bonding. The examined research includes single studies and reviews that use both qualitative and quantitative methods. Drawing on animal and human models, the effect of music on foetal behaviour and various possible biological, psychological and social mechanisms are discussed. The potential preventive effect of music-based interventions, approaches and practices and their possible use across different cultures are also considered. Overall, we highlight how music, employed in a variety of ways, may support perinatal mental health with the aim of stimulating more interest and research in this area.

## Introduction

“Music gives a soul to the universe, wings to the mind, flight to the imagination and life to everything.”―Plato

Music is ubiquitous, emotional, social and communicative (MacDonald et al. 2012a, b) and, in its most broad definition, present in every known culture (Wallin et al. 2000). Music’s medicinal value has been discussed and documented from as early as 4000 BC (Spintge and Dorh 1992), showing a long history of music and its relationship with health and well-being. Music has been called the ‘most under-utilised asset on the planet’ (House of Lords 2019), highlighting music’s potential to drive health and economic returns in the UK. A report from the World Health Organisation (WHO) has reviewed the global evidence linking the arts to health and well-being. It confirms that engaging with the arts has a positive influence on both mental and physical health from before birth to the end of life (Fancourt and Finn 2020). This descriptive overview, henceforth referred to as an overview, focuses on the potential of music-based interventions, approaches and practices to help support perinatal mental health.

There are several different types of music-based interventions, approaches and practices which may be helpful for supporting health and well-being. MacDonald’s conceptual model describes five overlapping music, health and well-being activities and practices: music therapy, music medicine, community music, music education and everyday uses of music (MacDonald 2013). Music therapy, a profession which has been producing research and developing their practice for more than 100 years (Edwards 2016), is distinct in its emphasis on the therapeutic relationship between a trained and licenced music therapist and the client who together work towards achieving a specific therapeutic goal. Community music focuses on providing the opportunity for musical engagement and its benefits within people’s local communities (e.g. community choirs or percussion classes). These types of interventions, activities and practices, while they may influence health outcomes, are different from music therapy in that they are led by community musicians as opposed to licenced music therapists. Music medicine is an approach where ‘prescribed music’ is used with a specific health outcome in mind. This typically involves a clinician choosing music for a patient to listen to while in the hospital or while receiving specific treatment. Music education focuses on the explicit development of conventional music skills. Everyday uses of music, while not a distinct practice, includes the passive and active ways people engage with music. Recent research has shown the positive effect this type of passive music listening can have on people’s health and well-being (DeNora 2000; Sloboda and O’Neill 2001).

While each of these interventions, approaches and practices is distinct, especially in their research, training and practice traditions, there is significant overlap in the theoretical basis behind their potential benefits within healthcare settings. For example, all draw on the similar psychological effects of music, including its ability to alter mood (Juslin and Västfjäll 2008; Lundqvist et al. 2009) and promote the formation of social bonds (Savage et al. 2020). For a more in-depth discussion of the distinctions and overlap between these different music-based interventions, activities and practices, see MacDonald (2013).

Engaging in music through various interventions, approaches and practices has been shown to be beneficial in reducing symptoms of depression and anxiety. For example, a Cochrane review (Aalbers et al. 2017) found that music therapy (involving both listening to and making music with a music therapist), as an additional intervention on top of usual treatment, reduced participants’ depression and anxiety symptoms when measured by both clinical assessment and self-report. Community group music interventions, such as drumming sessions (Fancourt et al. 2016), have also been found to improve anxiety and depression symptoms in mental health service users in the UK. A frequently studied music activity, used within community music and music therapy, is group singing (Clift 2012; Dingle et al. 2019; Heydon et al. 2020). Engaging in group singing has been shown to increase participants’ mental health and well-being, enjoyment, emotional state, sense of belonging and self-confidence (Williams et al. 2018). Group singing has also been shown to promote feelings of social closeness or social bonding (Freeman 2000; Dunbar 2012). Research on the impact of music-based interventions, approaches and practices on people’s health, mental health and well-being has become a growing area of research interest, inspiring a range of systematic reviews and books on the topic (see Clift 2012; MacDonald et al. 2012a; Aalbers et al. 2017; Daykin et al. 2018; Williams et al. 2018).

This article is an overview of selected research about music-based interventions, approaches applicable to perinatal mental health. The selected research includes single studies and reviews that use both qualitative and quantitative methods. While recognising the risk of conflating across different practices, methods or disciplines, we explicitly draw together examples of research from related but distinct evidence bases and disciplines (MacDonald et al. 2012a, b; Tsiris et al. 2016; Sanfilippo and Spiro 2017; Heydon et al. 2020). In fact, the insights that can be gained from taking an interdisciplinary perspective on the role of music in care contexts is the focus of a forthcoming book on musical care throughout the life course (Spiro and Sanfilippo, in press).

For the purposes of this article, this overview focuses on select areas of interest, including the use of music-based interventions, approaches and practices to reduce pain and anxiety during labour, to help reduce anxiety symptoms in pregnancy, to reduce symptoms of postnatal depression, to help foster stronger mother–infant bonding, as a potential preventative approach, and across cultures. Given this overview’s focus on perinatal mental health, areas beyond the scope of this paper which will not be covered include, for example, music therapy in the neonatal intensive care unit (Haslbeck 2012; Bieleninik et al. 2016; Shoemark and Ettenberger 2020) and the musical experience and development of infants (Trehub et al. 1997; de l’Etoile 2008; Trehub 2016). There are several systematic and narrative reviews on the use of music-based interventions, approaches and practices to support maternal health and mental health including, e.g. McKinney (1990), Terry and Terry (2012), Hollins Martin (2014), Corbijn van Willenswaard et al. (2017), Wulff et al. (2017), Parmar (2019), Yang et al. (2020) and Shimada et al. 2021. The purpose of this overview is to provide a broader view of this area than is possible in these more targeted reviews. The overarching aim is to stimulate interest and future research by providing examples of research across different practices and research disciplines.

## Benefits of music-based interventions, approaches and practices during the perinatal period

Listening to relaxing music has been investigated in many studies related to obstetrics. For example, Buglione et al. (2020), in a non-blinded randomised control trial, showed that listening to self-selected music over speakers, compared to no music, significantly reduced participants’ pain level and anxiety during labour when measured by self-report visual analogue scales (VAS). In a single-blind randomised control trial, Gönenç and Dikmen (2020) investigated the effect of a music and dance intervention on pain and fear during active labour. The 30-min dance and music intervention involved participants listening to three self-selected songs on headphones while being led by the researcher in dance moves involving moving and circulating the pelvis. This study (*n* = 93) found that dance and music, and listening to the music alone on headphones, significantly reduced self-reported pain and fear during the active phase of labour when compared to standard care. In their review, Wulff et al. (2017) provide other examples of the use of music-based interventions in obstetrics, including during caesarean section.

Lin et al. (2019) reviewed the effect of listening to music during pregnancy on symptoms of anxiety, evaluating 11 randomised control trials. Although the context (whether participants were at home or in hospital) and music choice (researcher versus self-chosen) varied across studies, a pooled meta-analysis showed that, overall, listening to music did reduce pregnant women’s symptoms of anxiety. The study was unable to determine whether the type of music (researcher versus self-chosen) had a significant influence on the effect size, and this remains to be clarified by future research. Additionally, the authors point out limitations, including small sample sizes, insufficient description of the musical material used and the predominant use of self-report measures. In another review of five randomised control trials which collectively included 1261 participants, Corbijn van Willenswaard et al. (2017) investigated both ‘active’ approaches, such as group music-making, and ‘passive’ approaches, such as music listening interventions. Both types of musical engagement were found to significantly reduce levels of maternal self-reported anxiety in pregnancy. However, there was no significant effect on self-reported general stress or pregnancy-specific stress. The authors also highlight that the methodological quality of the included studies was moderate to weak. Taken together, these studies suggest that active music-making and music listening may reduce anxiety symptoms in pregnancy; however, more rigours work should be done to understand what may make these interventions successful (e.g. participant vs researcher chosen music) and for what subgroups these types of interventions may be the most beneficial.

There are also studies investigating the effect of music-based interventions, approaches and practices for reducing depression symptoms in postnatal women, with mounting evidence indicating a potential benefit. Corey et al. (2019) conducted a qualitative study of both hospitalised antenatal and postnatal women who received a single bedside session with a credentialed music therapist. Participants were referred to music therapy to help reduce stress and anxiety, for emotional support, or for mother–baby bonding support. The music therapy sessions included tailored interventions and education in relaxation techniques. Music therapy was provided to 223 postpartum and 97 antepartum patients, and using manual thematic coding of repeated concepts, words and phrases, the study found that the programme was feasible and well-received. Patients reported feeling more relaxed, high satisfaction with the sessions and a stronger connection with their baby. The study also reported that health providers and staff were enthusiastic about the use of music therapy within their clinics.

Fancourt and Perkins (2018a) ran a three-arm randomised control trial which followed 134 women with symptoms of postnatal depression. In this study, they compared the effect of singing group sessions, creative play group sessions and usual care on participants’ scores on the Edinburgh Postnatal Depression Scale (EPDS). The singing group sessions and play group sessions were led by the same professional workshop leaders; they lasted for an hour and were held weekly for over 10 weeks. Participants in the singing group listened to songs sung by the leader, sung songs with their babies and created new songs. Participants in the creative play group engaged in sensory play with their babies through arts and crafts and playing games. This study found that women who participated in the singing group sessions had a greater reduction in symptoms compared with women who took part in the non-musical playgroup sessions. Alongside this trial, Perkins et al. (2018) also carried out a qualitative study. At the end of the programme, semi-structured interviews were conducted with 54 participants who took part in the singing group sessions. The participants reported several benefits, including an ‘authentic, social and multicultural creative experience’, enhanced ability to calm babies and immersive ‘me time’ for themselves. The group singing intervention also facilitated a sense of achievement and identity and enhanced the mother–infant bond.

Research across ethnomusicology, psychology and music therapy has also shown the potential benefit of music-based interventions to support the mother–infant relationship, usually through maternal singing. Singing acts as a key channel through which meaningful, sensitive and contingent interactions happen between the mother and infant. These musical interactions have been shown to enrich the bond between the mother and her child (Milligan et al. 2003). A study conducted in England found that out of a sample of 473 mothers between 1 and 9 months post-birth, 71% of mothers reported listening to music on a daily basis while 87% of mothers reported singing to their babies on a regular basis, with 59% singing to them daily (Fancourt and Perkins 2018b).

Lullabies and play songs are found in all known cultures (Trehub and Trainor 1998), making them an important musical source for intervention, approach and practice development around the perinatal period. These types of songs are usually sung in an infant-directed manner where the tempo is slower, the pitch variability higher and the rhythm exaggerated (Trainor et al. 1997). Some theories suggest that infant-directed singing is one behaviour that may have evolved with the aim of enhancing mother–infant bonding (Falk 2004), defined as the affective state of the mother towards the infant (Bicking Kinsey and Hupcey 2013). Infant-directed singing has also been shown to be effective in maintaining infants’ composure, delaying the onset of distress (Corbeil et al. 2016) and modulating their arousal levels (Shenfield et al., 2003).

A cohort study by Persico et al. (2017) investigated the effects of singing lullabies during pregnancy on mother–infant bonding, newborns’ behaviour and maternal stress. The researchers recruited 156 women at 24 weeks and split them into a singing class group and an antenatal class control group. The 14 singing classes, held weekly, involved the participants learning nine musicologist-selected lullabies together with a midwife. After 4 weeks, women were invited to choose one or two lullabies to continue singing at home. The control condition involved an antenatal class, led by the same midwife, without the inclusion of the lullabies. However, it is not clear what activities were included in this control condition. The study found a significant increase in postnatal bonding as measured by the self-report Mother–Infant Bonding Scale but no effect on prenatal attachment as measured by the Prenatal Attachment Inventory. They also found that women who participated in the singing sessions had significantly lower perceived maternal stress and participant-reported incidence of neonatal crying episodes and infantile colic.

Music-based interventions, approaches and practices that support the use of infant-directed singing can enhance mother–infant interactions and bonds postnatally. Research has shown that women with postnatal depression tend to sing more slowly and in a less emotionally expressive way compared with mothers without depression (de l’Etoile and Leider 2011). Within music therapy, research has suggested that interaction coaching, where a music therapist models infant-directed singing to a depressed mother, can help support the mother and the interactions with her infant (de l’Etoile 2006; O’Gorman 2007; de L’Etoile 2012). Additionally, a study by Van Puyvelde et al. (2014), conducted within an inpatient setting for the treatment of severe postnatal depression, investigated the potential of a 5-week musical intervention, led by two performing artists, for supporting mother and infant mutual engagement across four mother–infant dyads. The study found that the intervention, which included music and movement aspects, increased the frequency and duration of mutual engagement between the mother and her infant.

Taken together, studies across various disciplines have shown the potential of music-based interventions, approaches and practices in supporting women with postnatal depression and the relationship between the mother and her infant. However, differences in methodology, approach and population across these selected studies make it difficult to understand what aspects of, and in what contexts, music-based interventions, approaches and practices may be helpful.

Listening to music during pregnancy may also have a direct effect on the foetus, especially in the last trimester as hearing develops. Kafali et al. (2011) showed that the foetus demonstrated a higher number of movements and an accelerated heart rate during exposure to music. Newborn babies have been shown to remember the music the mother listened to at the end of pregnancy, as well as respond to their mother’s voice as heard in utero (Hepper 1991; Hepper et al. 1993). Granier-Deferre et al. (2011) showed that such auditory memory, shown by the heart rate response to the music they had been played in the womb, could last until at least 6 weeks after birth. While these studies present some evidence that music can affect foetal behaviour and present evidence of foetal auditory learning, there is no good evidence at the moment that being exposed to music in utero can increase the child’s cognitive or behavioural development. However, there is strong evidence that maternal stress, anxiety and depression during pregnancy can increase the risk for the future child for a range of neurodevelopmental problems (Glover et al. 2018), so it remains possible that exposure to music during pregnancy may improve child outcome via an effect on the mother’s mood.

## Potential mechanisms

Remarkably, listening to music has been shown to alter the cognitive development of the offspring in animal models. Rauscher et al. (1998) showed that exposing rats to Mozart from in utero to 2 months postnatally improved their ability to navigate a maze. Listening to white noise or a minimalist Philip Glass composition had no such effect. Chikahisa et al. (2006) showed that if mice were played Mozart in the perinatal period, it enhanced the learning performance and altered the brain-derived neurotrophic factor signalling in the adult offspring. Kim et al. (2013) found that exposure to music during pregnancy increased neurogenesis in the motor and somatosensory cortex of the rat pups. Similar effects on cognitive or behavioural outcomes have not been supported in human models (Jones and Zigler 2002).

Animal models have also found that exposure to music has the ability to reduce anxiety and depressive behaviour in offspring after experiencing of postnatal stress. Papadakakis et al. (2019) used a model of early maternal separation in rats. They showed that if they played Mozart to these animals between 2 weeks and 2.5 months postnatally, this reversed the behavioural effects of the maternal separation and increased dendritic spine density in the CA1 region of the hippocampus. Similar effects of music on behavioural outcomes in the offspring have been found in human models; however, evidence is limited. For example, the study discussed earlier found that infants whose mothers took part in a prenatal group that encouraged lullaby singing prenatally and postnatally cried less than those in the play group condition, as measured by self-report (Persico et al. 2017).

There is some evidence in human models that explore the potential biological, psychological and social mechanisms that may underlie the beneficial effects of music exposure and engagement on maternal mental health, specifically in the context of making music with others. Group music-making, either singing, dancing or playing instruments together, involves synchronisation where participants move together in time, creating a ‘collective convergence’ (Cross 2014). The synchronisation of movement by entraining to the beat and one another has been suggested as one potential mechanism to explain why group singing and music-making in general increases people’s feelings of social cohesion (Dunbar 2012; Tarr et al. 2015). Synchronisation in music-making has also been found to have other social effects such as increased trust, shared intentions, shared attention, prosocial behaviours, shared success and positive affect (Kirschner Sebastian and Tomasello 2010; Reddish et al. 2013; Weinstein et al. 2016; Savage et al. 2020). A potential biological mechanism to explain this effect is the activation of the endogenous opioid system, which includes β-endorphins and enkephalins which are known to foster and maintain social bonds (Machin and Dunbar 2011), increase your mood, as well as to reduce the sensation of pain. Recent studies in music psychology have explored this potential relationship and found that pain thresholds became raised after engaging in group singing (Tarr et al. 2014, 2015; Pearce et al. 2015; Weinstein et al. 2016).

Research has also focused on the psychoneuroimmunology effects of music (Fancourt et al. 2014). For example, in a recent three-armed randomised control trial (*n* = 743), researchers investigated the effects of listening to relaxing music, versus participation in a singing class led by a music therapist, compared with treatment as usual on maternal well-being and mother–infant bonding (Wulff et al. 2021). The participants in the singing group participated in two singing classes, conducted by a music therapist, who led the participants in children’s songs and lullabies accompanied by live guitar. Those in the music listening group were given a CD with classical, calm music without lyrics and a soothing calm beat for use at home. Participants were also free to choose to listen to other music which they found relaxing. They measured biological markers, using saliva samples, including measures of cortisol, alpha-amylase and oxytocin levels. They found that both the singing and listening intervention showed positive effects on stress, with a greater reduction of cortisol levels and an increase in oxytocin after either listing to music or taking part in the singing group. They also found that the singing group showed a larger reduction in cortisol compared to the music listening group and a greater increase in general self-efficacy and perceived closeness to the unborn child when measured with a visual analogue scale. However, they found no significant effects on depressive symptoms when using the EPDS, potentially due to low baseline scores in their sample.

In a cross-over within-subjects study with mothers with babies between 3 and 14 months, researchers investigated differences in engaging in 35-min group sessions, which involved either mother-infant singing or non-musical mother-infant round group activities on social closeness, affect and both psychological and biological markers of anxiety (Fancourt and Perkins 2018c). The workshops were led by a trained workshop leader and were held in groups of 8–10 mother–baby dyads. Using saliva samples, they measured cortisol, cortisone and dehydroepiandrosterone (DHEA) in 43 participants. The singing workshop involved a song to start the session and close the session, the use of melodic vocal patterns and the learning of simple calming songs. The non-music round circle activity had a similar format, but instead of using songs, participants used non-melodic vocal exercises and played and talked with their babies. In this study, they found that participants who took part in the singing group had a greater increase in perceptions of social closeness, positive affect and a greater reduction in negative affect and greater decreases in both psychological and biological markers of anxiety compared to those in the non-musical group. Together, these studies highlight some of the potential psychoneuroimmunolgical, psychological and social mechanisms which may explain the positive effect of music-based interventions on maternal mental health.

## Music-based interventions, approaches and practices as preventative

Studies concerning the possible effects of music-based interventions as a preventive tool are just beginning. A recent Lancet Commission on global mental health and sustainable development specifically aims to reframe the global mental health agenda by steering researchers away from focusing on the binary diagnostic approach and instead towards considering symptoms along a spectrum of severity (Patel et al. 2018). This general approach to mental health interventions is more reflective of the actual experience of people with mental disorders and highlights the importance of both treatment and prevention (Patel et al. 2018).

The potential preventative or protective effect of music engagement for maternal mental health has been explored by a study of a prospective cohort in England (Fancourt and Perkins 2018d). This study followed 395 new mothers from the third trimester until 6 months post-birth and measured how often they listened to music, their postnatal depression symptoms using the EPDS and their overall well-being using the short Warwick–Edinburgh Mental Well-being Scale. They found that listening to music during pregnancy was associated with higher levels of well-being and reduced symptoms of postnatal depression in the first 3 months post-birth. However, the association disappeared by 4–6 months post-birth. This study points to a potential protective effect of general music engagement; however, the potential preventative effect of music-based interventions, approaches and practices during the perinatal period has not been significantly investigated, indicating an important area for future research.

## Music-based interventions, approaches and practices across cultures

Music interventions, approaches and practices may be particularly appropriate in the many global contexts where there is a rich musical culture, but a scarcity of trained health professionals. Parmar (2019) reviewed the effects of music-based interventions and music therapy on maternal and foetal health outcomes during the antenatal period. This review, undertaken in India, highlighted the potential application of music-based interventions and music therapy for maternal mental health in non-Western cultures, especially in countries such as India, where music is already an integral part of the culture and traditions around healing.

There has been little research on the potential efficacy of music-based interventions for maternal mental health across various contexts, specifically in low-and-middle-income countries (LMICs), where a music-based approach might be particularly useful as it is non-stigmatising, low-cost and can be culturally embedded. In a recent feasibility study in The Gambia, West Africa, has been carried out (Sanfilippo et al. 2019, 2020) a brief intervention, Community Health Intervention through Musical Engagement (CHIME), was co-developed with local Kanyeleng groups Kanyeleng groups are historically comprised of women who are dealing or have dealt with infertility or child mortality and play an important role in community ceremonies, as well as public health campaigns, commissioned by the Ministry of Health and Social Welfare (McConnell, 2019a). The music performances of the Kanyeleng are highly entertaining and participatory, involving humour, singing, dancing, hand-clapping and percussion (McConnell 2019b). The intervention was delivered by local Kanyeleng groups over 6 weekly hour-long sessions with groups of pregnant women across four communities. It was also delivered universally, meaning pregnant the participants (n = 124) were recruited (from antenatal clinics) regardless of their levels of anxiety and depression symptoms. The intervention sessions involved the Kanyeleng groups leading group singing and dancing, drawing on local, well-known repertoire, but improvising new lyrics around themes of resilience, the importance of social support and some strategies to deal with the common psychological and physical challenges of pregnancy. In addition, the sessions included a traditional lullaby that the participants could draw on after their child was born. The general aim of the intervention was to create a socially supportive network and lift the women’s mood, facilitated by group music-making. The control group received standard antenatal care. The feasibility trial found that not only was CHIME feasible, enjoyable and culturally acceptable but also resulted in a significant reduction in symptoms of anxiety and depression in those who received the intervention compared to those who received standard care (Sanfilippo et al. 2020). While the potential impact of this work in The Gambia is evident, this research also has implications for research in other LMICs and other marginalised communities where poverty and other risk factors may exacerbate symptoms of maternal mental distress and the musical and cultural context affords this type of co-developed community-based approach.

## Conclusion

In conclusion, there is considerable evidence, albeit of varying quality, that music-based interventions, approaches and practices can support perinatal mental health in a variety of ways, including by helping reduce anxiety and pain during labour, anxiety symptoms during pregnancy and symptoms of postnatal depression. Music-based interventions, approaches and practices, especially those which involve infant-directed singing have also shown potential benefits for supporting mother–infant bonding. Potential biological, psychological and social mechanisms, including those related to the psychoneuroimmunological effects of music and the social effects of group music-making, have recently been investigated in perinatal populations. There has also been discussion around the potential preventative effect of these types of interventions and approaches, although, as of yet, very little research has been conducted. Co-developed interventions, which capitalise on existing music practices, may be especially applicable in places that have rich musical traditions but few trained mental health professions. They present a culturally-embedded, non-stigmatising and low-cost intervention to support maternal mental health. Overall, this overview presented a selected set of examples of research from a variety of disciplines with the aim of stimulating future investigation into music-based interventions, approaches and practices that support perinatal mental health.

## Data Availability

Not applicable.
